# Sex-specific risk factors associated with graves’ orbitopathy in Korean patients with newly diagnosed graves’ disease

**DOI:** 10.1038/s41433-023-02513-z

**Published:** 2023-04-11

**Authors:** Jooyoung Lee, Jinmo Kang, Hwa Young Ahn, Jeong Kyu Lee

**Affiliations:** 1 Department of Applied Statistics, Chung-Ang University, Seoul, Korea; 2 Department of Internal Medicine, Chung-Ang University College of Medicine, Seoul, Korea; 3 Department of Ophthalmology, Chung-Ang University College of Medicine, Seoul, Korea

**Keywords:** Risk factors, Thyroid diseases

## Abstract

**Objective:**

To assess sex-specific risk factors for Graves’ orbitopathy (GO) in newly diagnosed Graves’ disease (GD) patients.

**Methods:**

A retrospective cohort study was conducted using the National Health Insurance Service’s sample database, which consisted of 1,137,861 subjects from 2002 to 2019. The international classification of disease-10 codes was used to identify those who developed GD (E05) and GO (H062). A multivariable Cox proportional hazards model was used to estimate the effect of risk factors on GO development.

**Results:**

Among 2145 male and 5047 female GD patients, GO occurred in 134 men (6.2%) and 293 women (5.8%). A multivariable Cox regression model revealed that GO development was significantly associated with younger age (HR = 0.84, 95% CI = 0.73–0.98), low income (HR = 0.55, 95% CI = 0.35–0.86), and heavy drinking (HR = 1.79, 95% CI = 1.10–2.90) in men, and with younger age (HR = 0.89, 95% CI = 0.81–0.98), lower body mass index (HR = 0.55, 95% CI = 0.33–0.90), high total cholesterol (HR = 1.04, 95% CI = 1.01–1.06), hyperlipidaemia (HR = 1.37, 95% CI = 1.02–1.85), and lower statin dose (HR = 0.37, 95% CI = 0.22–0.62) in women. There was no association between smoking and GO development in both men and women.

**Conclusions:**

The risk factors for GO development were sex-dependent. These results show the need for more sophisticated attention and support considering sex characteristics in GO surveillance.

## Introduction

Graves orbitopathy (GO) is an autoimmune disorder mainly associated with Graves’ disease (GD) [1]. GO involves inflammatory changes in the orbital tissues and presents as variable signs and symptoms, including foreign body sensation, lid swelling, and exophthalmos. It is disfiguring, can produce visual disturbances, and seriously deteriorate the quality of life of patients. Although a more detailed understanding of the pathogenesis of GO has led to the investigation of various therapeutic drugs, the long-term adverse effects of GO have not yet been prevented. The identification and assessment of risk factors for the development of GO in GD patients will be of great help in alleviating patients’ anxiety and determining rational treatment strategies.

Previous studies have reported that insufficient control of hyperthyroidism [2], high thyroid-stimulating hormones receptor antibody levels (TSH-R-Ab) [3], smoking [4, 5], radioactive iodine (RAI) treatment [6, 7], and high cholesterol levels [8, 9] are established risk factors for the development of GO in patients with GD. However, few studies have been conducted to analyse these risk factors by sex. As is widely known, the prevalence of GO demonstrates a noteworthy, sex-specific characteristics—women are three to four times more likely to develop the disease than men [10]. However, men develop GO at an older age and suffer from worse clinical features despite a lower incidence [11]. Typically, sex differences in the incidence and clinical features suggest that the risk factors for GO may also differ between men and women. However, the aforementioned risks of GO development are mainly reported from studies on Western populations. Studies on the risk of GO occurrence in Asians have generally been conducted at the level of individual hospitals, and relatively few studies have been conducted at multiple centres or nationwide.

The purpose of this study was to identity sex-specific risk factors for GO development in patients with new-onset GD in Koreans. In the process of exploring risk factors for GO, we attempted to overcome hospital-level data limitation by conducting a nationwide, population-based, large-scale, cohort study.

## Materials and methods

### Data source

This is a retrospective cohort study using the National Health Insurance Service (NHIS)’s sample database, which consisted of 1,137,861 subjects from 2002 to 2019. The NHIS is the public medical insurance system managed by the Korean government, providing a compulsory insurance system for all residents in Korea. The NHIS also regularly provides free, standardized health checkups for all insured persons. This study was approved by the Institutional Review Board of Chung-Ang University Hospital (IRB No. 2107-035-19376), and informed consent was waived. This study adheres to the guidelines of the Declaration of Helsinki.

### Study population

**Fig. 1:**
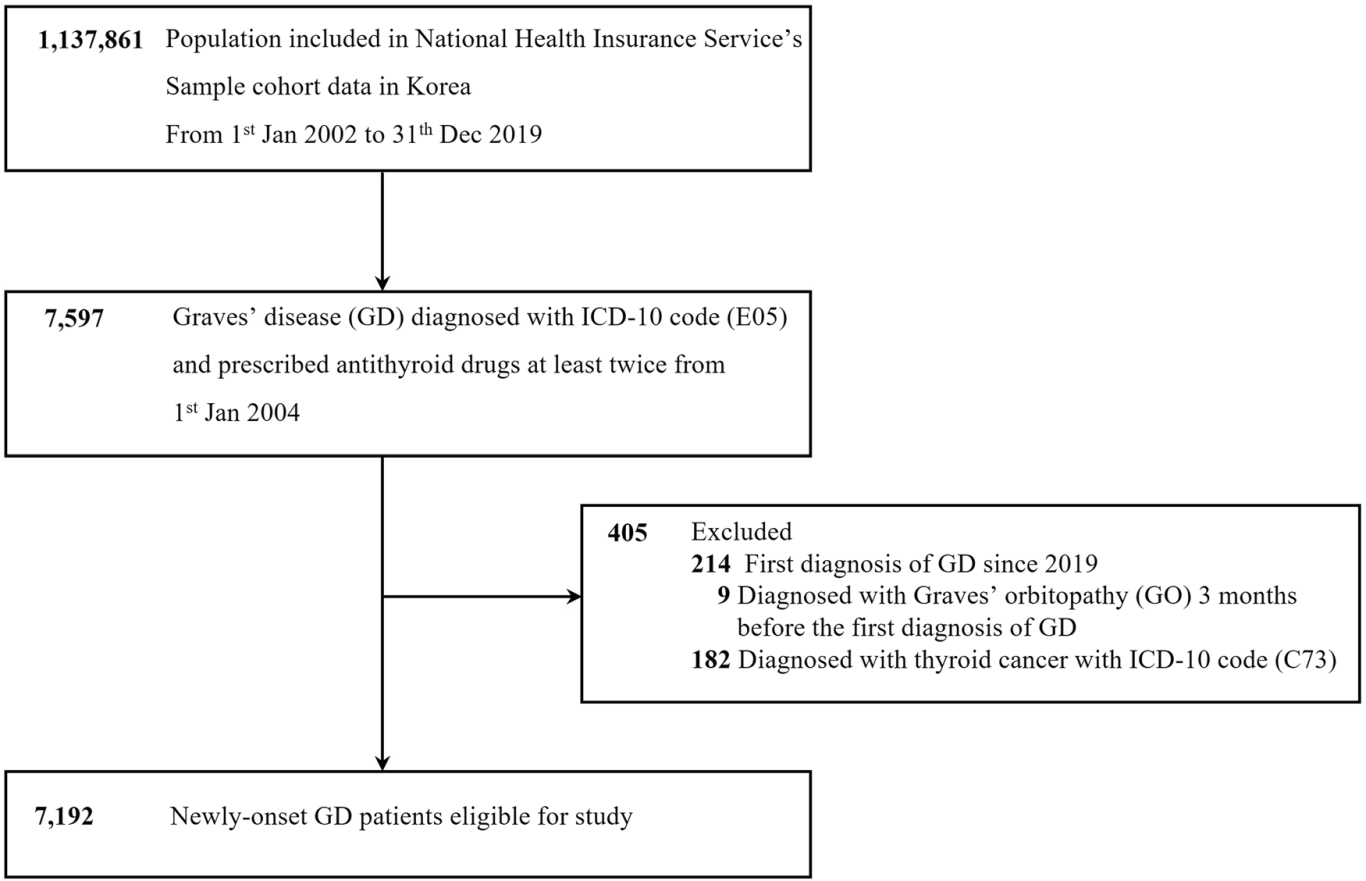
The flowchart of the study. A schematic overview illustrated the inclusion and exclusion criteria for study subjects.

New-onset GD patients were defined as subjects who were diagnosed at least twice with international classification of disease (ICD)-10 codes (E05) and who were prescribed a medication for hyperthyroidism for more than 180 days. Such new-onset GD patients were identified between 2004 and 2018. Those diagnosed with GD for the first time after 2019 or those diagnosed with GO before 3 months prior to being diagnosed with GD were excluded. Patients diagnosed with thyroid cancer with ICD-10 code (C73) were also excluded. A total of 7192 new-onset GD patients were eligible for our study (Fig. 1).

### Definition of outcomes and variables

GO patients were defined as those diagnosed with ICD-10 code (H062). Patents with active GO were defined as those who were prescribed intravenous steroids for more than 6 times within three months from the initial prescription of intravenous steroids since the GO diagnosis.

The health examination provided by NHIS includes survey responses and screening measurement values. Information on income level, residence, alcohol consumption, and smoking history was obtained at baseline through standardized, self-administered questionnaires collected within 3 years before the GD diagnosis. By income decile, those in the top ~30% were classified as high-income, those in the bottom ~30% as low-income, and the rest as middle-income. Residence was categorized into three categories: capital, metropolitan, and rural. Alcohol consumption was categorized into three categories: non-drinker, mild-to-moderate drinker (1–5 times a week and less than 7 drinks on any day per week), and heavy drinker (more than 6 times a week or more than or equal to 7 drinks on any day per week) [12]. Smoking status was classified as nonsmoker and ex- or current smoker. The body mass index (BMI) was calculated by dividing weight (kg) by height squared (m^2^). After an overnight fast, blood was drawn to measure blood glucose (mg/dL) and total cholesterol (mg/dL). The degree of control of GD could not be determined because thyroid function and TSH-R-Ab tests are not included in the health examination items of the NHIS.

We identified cases with comorbidities, including diabetes mellitus (DM), hyperlipidaemia, and autoimmune disease. DM was defined as subjects who were diagnosed at least twice with ICD-10 codes (E11–E14) or who had been prescribed oral hypoglycaemic agents or insulin. Hyperlipidaemia was defined as subjects diagnosed at least twice with ICD-10 codes (E78) or who had been prescribed lipid-lowering medication. Autoimmune disease was defined as subjects diagnosed with ICD-10 codes (rheumatoid arthritis M06, polymyalgia rheumatica M353, multiple sclerosis G35, type 1 diabetes mellitus E10, systemic lupus erythematous M32, sarcoidosis D86, Sjogren syndrome M350, myasthenia gravis G700, pernicious anaemia D51, autoimmune haemolytic anaemia D591, autoimmune adrenalitis D271). RAI treatment was defined as having a sodium iodide drug code. Thyroidectomy was defined as one of the following surgical codes: P4551, P4552, P4553, P4554, and P4561. In addition, among lipid-lowering drugs, statins were separately analysed. Exposure to statins was defined as having at least two prescriptions of atorvastatin, fluvastatin, lovastatin, pitavastatin, pravastatin, rosuvastatin, or simvastatin and was assessed at baseline and during the follow-up periods. The dose of each statin with an equivalent LDL cholesterol reduction effect for the various statins was calculated as 80 mg fluvastatin, 40 mg lovastatin, 2 mg pitavastatin, 40 mg pravastatin, 5 mg rosuvastatin, and 20 mg simvastatin based on 10 mg of atorvastatin [13, 14].

To address the aspect of time-varying comorbidities, history of RAI, and statin dose over time, time-dependent covariates for those variables were created for each month during the follow-up periods. We calculated the average daily statin dose per month by obtaining the average value of the daily atorvastatin equivalent statin dose (mg) each month. The codes used to identify disease and medications are available in the Supplementary Tables (Table S1 and Table S2).

### Statistical analysis

Descriptive statistics are shown as median (interquartile ranges) or mean ± standard deviation for continuous variables and percentage for categorical variables. The chi-square test or Fisher’s exact test was used for comparisons of categorical variables. Student’s *t* test or Mann–Whitney *U* test was used for comparisons of continuous variables at baseline and the end of follow-up. To summarize the change in statin dose for ever-statin users over the follow-up periods, the average daily statin doses per year were described and a linear regression was fitted for the statin doses to characterize a linear trend in the statin doses.

There were missing data in the health examination data. Multiple imputation using chained equations (MICE) were used to generate 10 imputed datasets with predictive mean matching for continuous data and logistic regression for binary data [15]. A multivariable Cox proportional hazards model was used to estimate the effects of risk factors on GO development, including baseline demographic, behaviour variables, and time-dependent variables of comorbidities, history of RAI, and statin doses with a time scale of a month. Subgroup analyses were performed according to sex to estimate the sex-specific hazard ratios (HRs) using multiply imputed data for each sex. To evaluate the effect modification by sex, we included interactions between sex and risk factors in the Cox models and performed a significance test for those interaction effects. In each analysis, the estimates were pooled using Rubin’s rule [16]. All GD patients included in the study were followed from the date of first diagnosis of GD to either the date of GO diagnosis, death, lost-to-follow-up, or December 31, 2019. For the subgroup analysis, active GO was assessed from the date of GO diagnosis to either the date of death, lost-to-follow-up, or December 31, 2019, for GO patients. We used the SAS software ver. 9.4 (SAS Institute, Cary, NC, USA) and R ver. 3.3 3 (R Foundation for Statistical Computing). For all analyses, *p* < 0.05 was considered statistically significant.

## Results

A total of 7192 GD patients were included in the cohort—2.4 times more women (70.2%) than men (29.8%). Among 2145 male and 5047 female GD patients, GO occurred in 134 men (6.2%) and 293 women (5.8%), indicating no sex-related difference in incidence. The median period of follow-up of the study participants was 94 (50–140) months for men and 102 (55–148) months for women.

Table 1 presents the characteristics of the study population according to sex. The median age of GD diagnosis was 40 (31–51) years for men and 41 (28–50) years for women among the GD patients with GO, which was younger than that of the GD patients without GO (*p* = 0.049, *p* < 0.001 respectively). The median time from the first GD diagnosis to the first GO diagnosis was 9 months for both men and women. At the time of diagnosis of GD, men had a higher incidence of GO in the low-income category and in the case of heavy drinkers, but there was no difference in women according to income or alcohol consumption. In women, higher BMI, comorbidity of DM, and taking statins were associated with a reduced risk of GO development (*p* = 0.024, *p* = 0.021, *p* = 0.014 respectively). Smoking was not associated with the risk of GO development in both men and women.

**Table 1:** Characteristics of study population by sex.

Variable	Men				Women			
	GO (*n* = 134)	Non-GO (*n* = 2011)	Overall (*n* = 2145)	*p* value	GO (*n* = 293)	Non-GO (*n* = 4754)	Overall (*n* = 5047)	*p* value
**At baseline**								
Age [median, IQR] (years)	40 [31; 51]	44 [34; 53]	43 [33; 53]	**0.049**	41 [28; 50]	44 [31; 55]	44 [31; 55]	**<0.001**
Region (%)				0.268				0.406
Capital	54 (40.3)	952 (47.3)	1006 (46.9)		151 (51.5)	2259 (47.5)	2410 (47.8)	
Metropolitan	33 (24.6)	416 (20.7)	449 (20.9)		56 (19.1)	999 (21.0)	1055 (20.9)	
Rural	47 (35.1)	643 (32.0)	690 (32.2)		86 (29.4)	1496 (31.5)	1582 (31.3)	
Income Grade (%)				**0.015**				0.398
Low	37 (27.8)	364 (18.6)	401 (19.1)		83 (28.5)	1191 (25.7)	1274 (25.9)	
Middle	40 (30.1)	772 (39.4)	812 (38.8)		98 (33.7)	1726 (37.3)	1824 (37.1)	
High	56 (42.1)	825 (42.0)	881 (42.1)		110 (37.8)	1709 (37.0)	1819 (37.0)	
Missing data	1	50	51		2	128	130	
Autoimmune Disease (%)				1.000				0.815
No	124 (92.5)	1861 (92.5)	1985 (92.5)		256 (87.4)	4184 (88.0)	4440 (88.0)	
Yes	10 (7.5)	150 (7.5)	160 (7.5)		37 (12.6)	570 (12.0)	607 (12.0)	
DM (%)				0.150				**0.021**
No	115 (85.8)	1616 (80.4)	1731 (80.7)		263 (89.8)	4023 (84.6)	4286 (84.9)	
Yes	19 (14.2)	395 (19.6)	414 (19.3)		30 (10.2)	731 (15.4)	761 (15.1)	
Hyperlipidaemia (%)				1.000				0.242
No	105 (78.4)	1,572 (78.2)	1677 (78.2)		239 (81.6)	3732 (78.5)	3791 (78.7)	
Yes	29 (21.6)	439 (21.8)	468 (21.8)		54 (18.4)	1022 (21.5)	1.076 (21.3)	
Statin user (%)				0.414				**0.014**
No	118 (88.1)	1822 (90.6)	1940 (90.4)		275 (93.9)	4238 (89.1)	4513 (89.4)	
Yes	16 (11.9)	189 (9.4)	205 (9.6)		18 (6.1)	516 (10.9)	534 (10.6)	
Smoking (%)				0.077				0.335
None	26 (23.6)	522 (32.2)	548 (31.7)		210 (92.1)	3615 (93.9)	3825 (93.8)	
Current or Ex-smoker	84 (76.4)	1098 (67.8)	1182 (68.3)		18 (7.9)	234 (6.1)	252 (6.2)	
Missing data	24	391	415		65	905	970	
Drinking (%)				**0.016**				0.354
None	27 (32.1)	517 (41.8)	544 (41.2)		102(71.3)	1879 (76.6)	1981 (76.3)	
Mild to Moderate	25 (29.8)	421 (34.0)	446 (33.8)		35 (24.5)	486 (19.8)	521 (20.0)	
Heavy	32 (38.1)	299 (24.2)	331 (25.0)		6 (4.2)	89 (3.6)	95 (3.7)	
Missing data	50	774	824		150	2300	2450	
BMI [median, IQR]	23.4 [21.9; 25.0]	23.3 [21.4; 25.4]	23.3 [21.5; 25.4]	0.964	21.9 [20.1; 24.3]	22.6 [20.6; 24.8]	22.5 [20.6; 24.8]	**0.024**
Missing data	50	751	801		147	2251	2398	
Total cholesterol (mg/dL) [median, IQR]	175.0 [156.0; 205.0]	173.0 [146.0; 202.0]	173.5 [146.0; 202.0]	0.309	181.0 [152.0; 203.0]	179.0 [152.0; 209.0]	179.0 [152.0; 208.0]	0.875
Missing data	50	759	809		147	2268	2415	
FBS (mg/dL) [median, IQR]	98.0 [87.5; 108.5]	97.0 [88.0; 107.0]	97.0 [88.0; 107.5]	0.793	95.0 [85.0; 103.0]	94.0 [86.0; 103.0]	94.0 [86.0; 103.0]	0.965
Missing data	50	751	801		147	2252	2399	
At the end of follow-up								
Autoimmune disease (%)				0.648				**0.016**
No	121 (90.3)	1782 (88.6)	1903 (88.7)		251 (85.7)	3787 (79.7)	4038 (80.0)	
Yes	13 (9.7)	229 (11.4)	242 (11.3)		42 (14.3)	967 (20.3)	1009 (20.0)	
DM (%)				**0.010**				**<0.001**
No	104 (77.6)	1335 (66.4)	1439 (67.1)		246 (84.0)	3406 (71.6)	3652 (72.4)	
Yes	30 (22.4)	676 (33.6)	706 (32.9)		47 (16.0)	1348 (28.4)	1395 (27.6)	
Hyperlipidaemia (%)				**<0.001**				**<0.001**
No	87 (64.9)	965 (48.0)	1052 (49.0)		218 (74.4)	2405 (50.6)	2623 (52.0)	
Yes	47 (35.1)	1046 (52.0)	1093 (51.0)		75 (25.6)	2349 (49.4)	2424 (48.0)	
Statin user (%)*				**<0.001**				**<0.001**
Non-statin user	116 (86.8)	1380 (68.6)	1496 (69.7)		276 (94.2)	3217 (67.7)	3493 (69.2)	
Statin user	18 (13.4)	631 (31.4)	649 (30.3)		17 (5.8)	1537 (32.3)	1554 (30.8)	
Thyroidectomy (%)				1.000				1.000
No	133 (99.3)	2004 (99.7)	2137 (99.6)		290 (99.0)	4712 (99.1)	5002 (99.1)	
Yes	1 (0.7)	7 (0.3)	8 (0.4)		3 (1.0)	42 (0.9)	45 (0.9)	
RAI (%)				0.912				0.748
No	126 (94.0)	1,878 (93.4)	2004 (93.4)		276 (94.2)	4447 (93.5)	4723 (93.6)	
Yes	8 (6.0)	133 (6.6)	141 (6.6)		17 (5.8)	307 (6.5)	324 (6.4)	
Period from GD to GO	9 [2; 27]				9 [2; 30]			
[median, IQR] (months)								

*GO* Graves’ orbitopathy, *n* number, *IQR* interquartile range, *DM* diabetes mellites, *BMI* body mass index, *FBS* fasting blood sugar, *RAI* radioactive iodin.

*Statin user assessed during the follow-up periods. Bold type denotes statistical significance (*p* < 0.05).

**Fig. 2:**
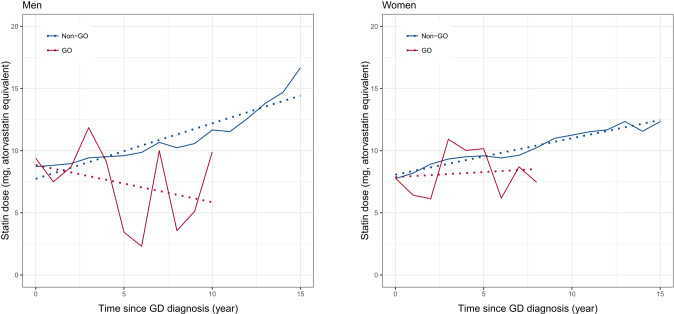
Changes in the average daily statin doses per year for ever-statin users according to GO development and sex. The solid lines represent mean trajectories of the average daily statin doses (mg) per year and the dotted lines represent the fitted linear trajectories.

To evaluate how the statins are related to GO development, changes in the statin dose over time were compared in both men and women. In both men and women, the statin dose continued to increase in GD patients without GO and also tended to increase in female GD patients with GO. In contrast, the statin dose tended to decrease over time in men with GO. Moreover, both men and women patients with GO showed a severe fluctuation in statin dose compared to patients without GO (Fig. 2).

**Fig. 3:**
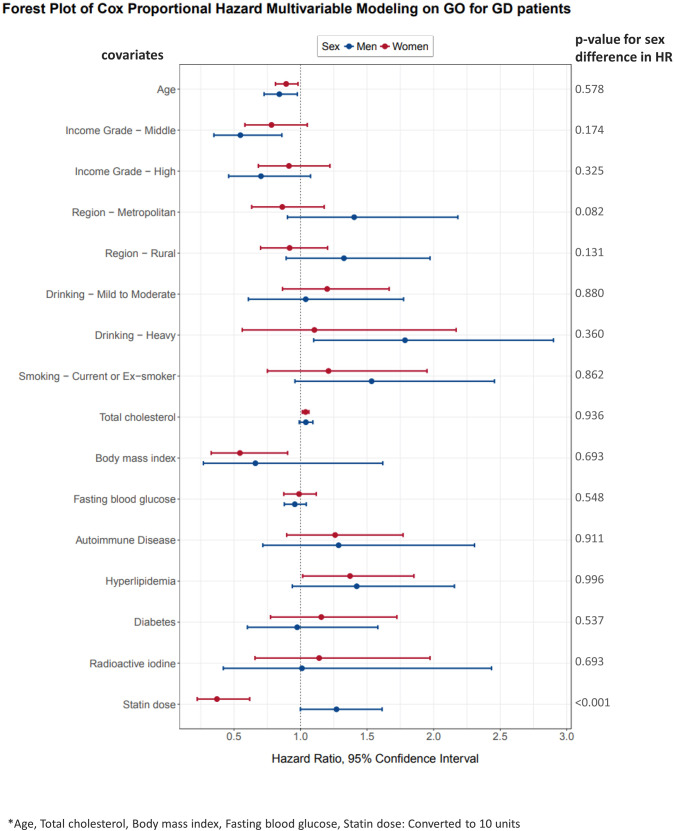
Hazard ratios and confidence intervals of Graves’ orbitopathy risk according to sex displayed in a Forest plot. Only the statin dose showed a significant difference in HRs between men and women.

In a multivariable Cox regression model, younger age (HR = 0.84, 95% CI = 0.73–0.98, *p* = 0.022), low income (HR = 0.55, 95% CI = 0.35–0.86, *p* = 0.009), heavy drinking (HR = 1.79, 95% CI = 1.10–2.90, *p* = 0.019) were significantly associated with GO development in men, while younger age (HR = 0.89, 95% CI = 0.81–0.98, *p* = 0.018), lower BMI (HR = 0.55, 95% CI = 0.33–0.90, *p* = 0.019), high total cholesterol (HR = 1.04, 95% CI = 1.01–1.06, *p* = 0.003), and hyperlipidaemia (HR = 1.37, 95% CI = 1.02–1.85, *p* = 0.039) were associated with GO development in women. In women who were satin users, a higher statin dose was significantly associated with a reduced risk of GO development (HR = 0.37, 95% CI = 0.22–0.62, *p* < 0.001) (Table 2). The HRs of individual risk factors calculated by the multivariable regression analysis were displayed as a Forest plot according to the sex of the patients; only the statin dose showed a significant difference in HRs between men and women (Fig. 3 and Table S3).

**Table 2:** Cox proportional hazard model analysis.

Variable	Men				Women			
	Estimate	SE	HR [95% CI]	*p* value	Estimate	SE	HR [95% CI]	*p* value
Age*	−0.17	0.08	0.84 [0.73, 0.98]	0.022	−0.11	0.05	0.89 [0.81, 0.98]	0.018
Region								
Capital	reference		1.00		reference		1.00	
Metropolitan	0.34	0.23	1.40 [0.90, 2.18]	0.132	−0.15	0.16	0.86 [0.63, 1.18]	0.353
Rural	0.28	0.20	1.33 [0.89, 1.97]	0.163	−0.09	0.14	0.92 [0.70, 1.20]	0.535
Income Grade								
Low	reference		1.00		reference		1.00	
Middle	−0.60	0.23	0.55 [0.35, 0.86]	0.009	−0.25	0.15	0.78 [0.58, 1.05]	0.102
High	−0.35	0.22	0.70 [0.46, 1.08]	0.104	−0.09	0.15	0.91 [0.68, 1.22]	0.539
Drinking								
None	reference		1.00		reference		1.00	
Mild to Moderate	0.04	0.27	1.04 [0.61, 1.77]	0.888	0.18	0.17	1.20 [0.87, 1.67]	0.271
Heavy	0.58	0.25	1.79 [1.10, 2.90]	0.019	0.10	0.34	1.10 [0.56, 2.17]	0.770
Smoking								
None	reference		1.00		reference		1.00	
Current or Ex-smoker	0.43	0.24	1.53 [0.96, 2.46]	0.074	0.19	0.24	1.21 [0.75, 1.95]	0.432
BMI*	−0.41	0.44	0.66 [0.27, 1.62]	0.354	−0.61	0.26	0.55 [0.33, 0.90]	0.019
Total cholesterol*	0.04	0.03	1.04 [0.99, 1.09]	0.117	0.04	0.01	1.04 [1.01, 1.06]	0.003
FBS*	−0.04	0.04	0.96 [0.88, 1.04]	0.316	−0.01	0.06	0.99 [0.87, 1.12]	0.852
Autoimmune disease								
No	reference		1.00		reference		1.00	
Yes	0.25	0.30	1.29 [0.72, 2.31]	0.398	0.23	0.17	1.26 [0.90, 1.77]	0.184
Hyperlipidaemia								
No	reference		1.00		reference		1.00	
Yes	0.35	0.21	1.42 [0.94, 2.16]	0.098	0.32	0.15	1.37 [1.02, 1.85]	0.039
DM								
No	reference		1.00		reference		1.00	
Yes	−0.03	0.25	0.98 [0.60, 1.58]	0.918	0.15	0.20	1.16 [0.78, 1.72]	0.476
RAI								
No	reference		1.00		reference		1.00	
Yes	0.01	0.45	1.01 [0.42, 2.44]	0.980	0.13	0.28	1.14 [0.66 1.97]	0.641
Statin dose*	0.24	0.12	1.27 [1.00, 1.61]	0.050	−0.99	0.26	0.37 [0.22, 0.62]	<0.001

*SE* standard error, *HR* hazard ratio, *CI* confidence interval, *BMI* body mass index, *FBS* fasting blood sugar, *DM* diabetes mellitus, *RAI* radioactive iodine.

*Converted to 10 units.

Statin dose: mg, atorvastatin equivalent.

There were 60 (14.1%) patients with active GO out of 427 patients with GO. There was no significant difference in the occurrence of active GO according to sex (*p* = 0.616). In patients who progressed to active GO, the median ages at the time of GD diagnosis were 49 (44–59) years for men and 47 (38–53) years for women, which were significantly older than those of inactive GO patients (*p* = 0.005, *p* = 0.009 respectively). In men, the proportion of statin users at the time of GD diagnosis was significantly higher in active GO patients than in inactive GO patients (Table S4).

## Discussion

GO is known to have a higher prevalence in women than in men. In this study, GD was 2.4 times more common in women than in men, but the incidence of GO in GD patients was similar in both sexes—6.2% in men, and 5.8% in women. The higher incidence of GO in women may be attributed to the higher prevalence of GD in women and the shared pathogenesis of both diseases. Although there have been studies on the effects of sex hormones on thyroid function and sex-related differences in iodine intake in thyroid disease [17, 18], such sex-related differences have not been studied as risk factors for developing GO. Therefore, we tried to identify sex-specific risk factors for GO through a large population-based cohort study. As a result, our regression model showed that younger age, low income, and heavy drinking were associated with GO development in men. In women, younger age, high serum total cholesterol, and lower BMI at the time of GD diagnosis, and hyperlipidaemia after GD diagnosis were associated with GO development. In addition, higher statin doses in women statin users after GD diagnosis were significantly associated with a reduced risk of GO development. Interestingly, in women, factors related to metabolic disorder, such as hyperlipidaemia, hypercholesterolemia, and statin use, were identified as the primary risk factors, while in men, sociobehavioural factors were associated with GO development.

Previous studies have reported that high levels of total and LDL-cholesterol correlated with the presence of GO and the clinical activity score [8, 9]. Our results also indicated that the comorbidity of hyperlipidaemia and serum total cholesterol were significantly associated with GO development in women. Both were found to be insignificant or even to reduce GO risk in a simple comparison, but the combination in a cox regression model revealed that there was a significant association with GO development in women. These conflicting results between the simple comparison and the regression model may be due to longer follow-up in patients without GO than in patients with GO whose follow-up was discontinued after the occurrence of GO according to our study protocol. Usually, the incidence of hyperlipidaemia gradually increases with age, which may be the case in patients without GO who had a longer follow-up period in our study. The confounding effect of the simultaneous use of statins to treat hyperlipidaemia may also be another reason. Statins were negatively correlated with GO development in our study. Hyperlipidaemia and statin use in men did not show any significant association with GO risk in the regression model, despite their association in a simple comparison. Total cholesterol can also have a positive correlation with age. In this study, the characteristics of women patients with GO with younger age and lower BMI may have influenced the reverse result in simple comparison compared to the regression model with a significant relationship between total cholesterol levels and GO development. The mechanism by which hypercholesterolemia or hyperlipidaemia induces GO development is not clear, but it may be associated with systemic, chronic inflammation caused by impaired lipid metabolism.

As hypercholesterolemia emerges as a risk factor for GO, there is growing interests in determining whether GO development can be reduced by statin use. Statins have anti-inflammatory effects and are expected to inhibit the cascade of inflammatory cell and cytokine activation in GO [19, 20]. Moreover, the inhibition of adipogenesis by statins may suppress GO symptoms. In this study, after confirming the preventive effect of statins on GO development, the equivalent statin dose was calculated to determine the differences in dose-response according to sex. Higher doses of statins were associated with a higher GO risk in men (*p* = 0.05) but a significantly lower GO risk in women (*p* < 0.001). Considering that statin dose tended to increase over time in GD patients without GO and in women GO patients (Fig. 2), women GD patients with good adherence to statin use seemed to have a much lower risk of developing GO than statin non-users, suggesting the protective effect of statin use. However, it is difficult to explain the relationship between statin dose and the risk of GO in men, although compliance may be one of the reasons, considering that statin dose decreases with time in male GO patients. Therefore, the apparent sex-related differences in statin doses observed in the Forest plots may reflect differences in physiological response or may be due to differences in drug compliance between the sexes.

In men, low income and heavy drinking were found to be associated with the occurrence of GO. A previous study revealed that low social grade and higher social deprivation were associated with more severe GO [21]. People with low socioeconomic status may have fewer opportunities to seek appropriate treatment in hospitals or may be more reluctant to receive treatments. Our study did not perform the thyroid function test for these subjects, but it is likely that GD may not be well controlled in these patients, causing an acceleration in GO occurrence. As is widely known, uncontrolled GD is an important risk factor for GO development. Diet may also be important. Oxidative stress is known to result in the production of free radicals, leading to the proliferation and inflammation of orbital fibroblasts in GO [22]. Patients with lower incomes may have lower intakes of antioxidants in the food they consume [21]. Heavy drinking is also estimated to worsen this lack of antioxidants [23]. It is difficult to explain why these socioeconomic factors are particularly related to the occurrence of GO in men rather than women. Our findings highlight the importance of improving access to high-quality care for these patients.

Smoking is the risk factor most consistently associated with the onset and severity of GO [24]. However, our study did not find a significant association between smoking and GO development in both men and women. In men, 76.4% of GO patients were past or current smokers with high smoking rates, but it did not reach the statistical significance (*p* = 0.077). Presumably, high proportion (68.3%) of smokers in GD patients contributed to the failure of deriving statistical significance. In contrast, in women, only 6.2% of the GD patients were smokers, so it is estimated that the number was insufficient to analyse the effect of smoking on GO development. The social environment in which smoking is more widespread in men than women seems to have influenced the results. Alternatively, these results could be because only the smoking status, but not the amount or period of smoking, was analysed.

RAI treatment is a well-known risk factor for GO deterioration [25] and is therefore, administered more cautiously to GD patients with prophylactic oral corticosteroids. Our study did not show any significant association between RAI treatment and GO development. The proportion of patients with GD who received RAI treatment in this study was relatively low, less than 7%. However, Korean doctors give priority to antithyroid drug treatment for the treatment of GD, with ~8% of patients receiving RAI treatment [26]. It is possible that the low RAI treatment rate contributed to the relatively low incidence of GO. Changes in clinical practice to the concomitant use of steroids during RAI treatment in patients with high risk of GO are also presumed to be responsible for the relatively low GO occurrence. Thyroidectomy was also not associated with GO development in GD patients. Several studies have reported that thyroidectomy is neutral to the natural course of GO [27, 28].

Abnormal levels of serum thyroid hormone are associated with GO occurrence, and proper management of thyroid dysfunction is paramount. Insufficient regulation or rapid changes in thyroid function affect GO progression. Serum TSH-R-Ab helps in assessing and predicting the response of antithyroid treatment, but it has also been reported as an important risk factor related to the severity and activity of GO [29]. However, this study was conducted based on NHIS’s claims data, and detailed serum measurements could not be analysed because NHIS does not collect any data on thyroid function tests or TSH-R-Ab titer. Thus, the lack of data on the control of GD, which is an important risk factor for GO, is a limitation of this study. Further research is needed to determine whether GD control is affected by sex.

Only older age in both sexes and statin use in men were associated with the progression to active GO in this study, but it was difficult to perform regression analysis due to the small numbers of relevant risk factors and patients with active GO. Previous studies have reported that older age, male sex, and smoking can affect the severity of GO [11, 30]. Although smoking did not show any statistical significance, our results demonstrated that the older age was related to the progression of active GO in both men and women.

This study has several limitations. First, this was a retrospective cohort study, where our data could only suggest the risk factors associated with GO development: the causative relationship could not be predicted. Second, since our study used insurance data consisting of diagnostic codes, there might be a possibility of misdiagnosis. In our study, GO occurred in ~6% of GD patients, which was lower than in previous studies [31]. Patients with mild GO may have been excluded as GO was defined using only the diagnostic code, and it is possible that the incidence of GO was underestimated. Finally, as our subjects consisted mostly of Koreans, our results might not be applicable to other ethnicities. However, the present study was a population-based, nationwide, large-scale cohort study with many advantages to offset its limitations. Moreover, time-dependent analyses for time-varying variables such as statin use and comorbidities were performed to minimize guarantee-time bias.

In conclusion, this is the first study to consider sex-related characteristics as risk factors for GO development. We found that younger age, low income, and heavy drinking were associated with GO development in men, while younger age, lower BMI, high total cholesterol, hyperlipidaemia, and lower statin doses in statin users among women were associated with GO development. Further studies are necessary to determine the causal relationship between these risks and GO development and to evaluate whether management of these risks modifies GO progression.

Supplementary information is available at Eye’s website.

### Summary

#### What was known before

- Graves’ orbitopathy (GO) is three to four times more likely to develop in women than in men. Uncontrolled hyperthyroidism, high TSH receptor antibody, smoking, radioactive iodine treatment, and high cholesterol levels have been reported as risk factors for the development of GO in patients with Graves’ disease (GD).

#### What this study adds

- GO development was associated with younger age, low income, and heavy drinking in men, and with younger age, low BMI, high total cholesterol, hyperlipidaemia, and lower doses of statin in statin users in women. GO patients had more fluctuation in statin dose than GD patients without GO in both men and women, and women GD patients with good adherence to statin use had a lower risk of developing GO than statin non-users.

### Supplementary information

Table S1

Table S2

Table S3

Table S4

## Data Availability

The data for this study is available from the corresponding author on reasonable request.
