# Supplementary material for: Sex-specific risk factors associated with graves’ orbitopathy in Korean patients with newly diagnosed graves’ disease
Source: Eye (Lond). 2023 Apr 11;37(16):3382–91. doi: 10.1038/s41433-023-02513-z (PMC10630462; doi:10.1038/s41433-023-02513-z)
Supplement: Supplementary file 1 — Table S1 [file 41433_2023_2513_MOESM1_ESM.docx]

Table S1. ICD-10 codes of diagnoses used for defining the study population, comorbidities, and outcomes

| Diagnosis | ICD-10 codes^¶^ |
| --- | --- |
| GD | E05 |
| Thyroid cancer | C73 |
| Autoimmune disease | D271, D510, D591, D86, E10, G35, G700, L80, M06, M32, M350, M353 |
| Hyperlipidemia | E78 |
| Diabetes mellitus | E11, E12, E13, E14, E15, R73 |
| GO | H062 |

^¶^International Classification of Disease, 10th revision by the World Health Organization

GD, Graves’ disease; GO, Graves’ orbitopathy
