# Supplementary material for: Sex-specific risk factors associated with graves’ orbitopathy in Korean patients with newly diagnosed graves’ disease
Source: Eye (Lond). 2023 Apr 11;37(16):3382–91. doi: 10.1038/s41433-023-02513-z (PMC10630462; doi:10.1038/s41433-023-02513-z)
Supplement: Supplementary file 2 — Table S2 [file 41433_2023_2513_MOESM2_ESM.docx]

Table S2. Medications of diagnoses used in the study

| Diagnosis | Medications |
| --- | --- |
| Graves’ disease | carbimazole, methimazole, propylthiouracil |
| Hyperlipidemia | atorvastatin, bezafibrate, ciprofibrate, clinofibrate, etofibrate, ezetimibe, fenofibrate, fenofibric acid, fluvastatin, gemfibrozil, lovastatin, micronized fenofibrate, omega-3-acid ethyl esters90, omega-3-acid triglycerides, pitavastatin, pravastatin, rosuvastatin, simvastatin |
| Diabetes mellitus | acarbose, albiglutide, alogliptin, anagliptin, dapagliflozin, dulaglutide, empagliflozin, evogliptin, exenatide, gemigliptin, glibenclamide, gliclazide, glimepiride, glipizide, human insulin, insulin aspart, insulin degludec, insulin detemir, insulin glulisine, insulin lispro, ipragliflozin, linagliptin, lixisenatide, lobeglitazone, metformin hydrochloride, miglitol, mitiglinide calcium hydrate, nateglinide, pioglitazone, repaglinide, rosuvastatin, saxagliptin, sitagliptin, teneligliptin, vildagliptin, voglibose |
| Active Graves’ orbitopathy | methylprednisolone succinate sodium |
