# Supplementary material for: Sex-specific risk factors associated with graves’ orbitopathy in Korean patients with newly diagnosed graves’ disease
Source: Eye (Lond). 2023 Apr 11;37(16):3382–91. doi: 10.1038/s41433-023-02513-z (PMC10630462; doi:10.1038/s41433-023-02513-z)
Supplement: Supplementary file 3 — Table S3 [file 41433_2023_2513_MOESM3_ESM.docx]

Table S3. Cox Proportional Hazard Model Analysis including Interactions between Risk Factors and Sex

| Variable | Estimate | SE | HR [95% CI] | *p* value |
| --- | --- | --- | --- | --- |
| Sex  Male  Female | reference  0.20 | 1.21 | 1.00  1.22 [0.11, 13.60] | 0.869 |
| Age | -0.17 | 0.08 | 0.84 [0.72, 0.99] | **0.033** |
| Region  Capital  Metropolitan  Rural | reference  0.32  0.28 | 0.23  0.20 | 1.00  1.38 [0.89, 2.15]  1.32 [0.89, 1.96] | 0.150  0.173 |
| Income Grade  Low Middle  High | reference  -0.61  -0.35 | 0.23  0.21 | 1.00  0.54 [0.35, 0.86]  0.71 [0.47, 1.08] | **0.008**  0.105 |
| Drinking  None  Mild to Moderate  Heavy | reference  0.12  0.71 | 0.24  0.27 | 1.00  1.12 [0.70, 1.80]  2.04 [1.20, 3.45] | 0.630  **0.009** |
| Smoking  None  Current or Ex-smoker | reference  0.28 | 0.21 | 1.00  1.33 [0.88, 2.01] | 0.176 |
| BMI* | -0.44 | 0.44 | 0.64 [0.26, 1.57] | 0.322 |
| Total cholesterol* | 0.03 | 0.03 | 1.04 [0.98, 1.09] | 0.204 |
| FBS* | -0.03 | 0.04 | 0.97 [0.90, 1.04] | 0.365 |
| Autoimmune disease  No  Yes | reference  0.27 | 0.30 | 1.00  1.30 [0.73, 2.34] | 0.375 |
| Hyperlipidemia  No  Yes | reference  0.33 | 0.22 | 1.00  1.39 [0.91, 2.12] | 0.132 |
| DM  No  Yes | reference  -0.06 | 0.24 | 1.00  0.94 [0.59, 1.51] | 0.802 |
| RAI  No  Yes | reference  -0.05 | 0.45 | 1.00  0.96 [0.40, 2.30] | 0.919 |
| Statin dose* | 0.25 | 0.12 | 1.28 [1.00, 1.63] | **0.046** |
| Female: Age | 0.05 | 0.09 | 1.05 [0.88, 1.26] | 0.578 |
| Female: Region  Capital  Metropolitan  Rural | reference  -0.48  -0.37 | 0.28  0.24 | 1.00  0.62 [0.36, 1.06]  0.69 [0.43, 1.12] | 0.082  0.131 |
| Female: Income Grade  Low Middle  High | reference  0.37  0.26 | 0.27  0.26 | 1.00  1.45 [0.85, 2.48]  1.29 [0.78, 2.14] | 0.174  0.325 |
| Female: Drinking  None  Mild to Moderate  Heavy | reference  -0.05  -0.41 | 0.31  0.44 | 1.00  0.95 [0.52, 1.77]  0.67 [0.27, 1.62] | 0.880  0.360 |
| Female: Smoking  None  Current or Ex-smoker | reference  -0.05 | 0.30 | 1.00  0.95 [0.52, 1.72] | 0.862 |
| Female: BMI* | -0.17 | 0.43 | 0.84 [0.36, 1.97] | 0.693 |
| Female:Total cholesterol* | 0.00 | 0.03 | 1.00 [0.94, 1.06] | 0.936 |
| Female: FBS* | 0.03 | 0.05 | 1.03 [0.94, 1.13] | 0.548 |
| Female: Autoimmune disease  No  Yes | reference  -0.04 | 0.35 | 1.00  0.96 [0.49, 1.90] | 0.911 |
| Female: Hyperlipidemia  No  Yes | reference  0.00 | 0.26 | 1.00  1.00 [0.60, 1.67] | 0.996 |
| Female: DM  No  Yes | reference  0.19 | 0.31 | 1.00  1.21 [0.66, 2.20] | 0.537 |
| Female: RAI  No  Yes | reference  0.21 | 0.53 | 1.00  1.23 [0.44, 3.45] | 0.693 |
| Female: Statin dose* | -1.22 | 0.28 | 0.30 [0.17, 0.52] | **<0.001** |

SE, standard error; HR, hazard ratio; CI, confidence interval; BMI, body mass index; FBS, fasting blood sugar; DM, diabetes mellitus; RAI, radioactive iodine

*Converted to 10 units

Statin dose: mg, atorvastatin equivalent
