# Supplementary material for: Sex-specific risk factors associated with graves’ orbitopathy in Korean patients with newly diagnosed graves’ disease
Source: Eye (Lond). 2023 Apr 11;37(16):3382–91. doi: 10.1038/s41433-023-02513-z (PMC10630462; doi:10.1038/s41433-023-02513-z)
Supplement: Supplementary file 4 — Table S4 [file 41433_2023_2513_MOESM4_ESM.docx]

Table S4. Sex-specific characteristics of patients with active Graves’ orbitopathy

| Variable | Men | | | | Women | | | |
| --- | --- | --- | --- | --- | --- | --- | --- | --- |
|  | Inactive GO  (n = 113) | Active GO  (n = 21) | Overall  (n = 134) | *p* value | Inactive GO  (n = 254) | Active GO  (n = 39) | Overall  (n = 293) | *p* value |
| **At baseline** |  |  |  |  |  |  |  |  |
| Age [median, IQR] (years) | 39 [30; 50] | 49 [44; 59] | 40 [31; 51] | **0.005** | 39 [27; 49] | 47 [38; 53] | 41 [28; 50] | **0.009** |
| Region (%)  Capital  Metropolitan  Rural | 46 (40.7)  26 (23.0)  41 (36.3) | 8 (38.1)  7 (33.3)  6 (28.6) | 54 (40.3)  33 (24.6)  47 (35.1) | 0.578 | 131 (51.6)  46 (18.1)  77 (30.3) | 20 (51.3)  10(25.6)  9 (23.1) | 151 (51.5)  56 (19.1)  86 (29.4) | 0.448 |
| Income Grade (%)  Low  Middle  High  Missing data | 31 (27.7)  34 (30.4)  47 (41.9)  1 | 6 (28.6)  6 (28.6)  9 (42.8)  0 | 37 (19.1)  40 (38.8)  56 (42.1)  1 | 0.987 | 69 (27.4)  87 (34.5)  96 (38.1)  2 | 14 (35.9)  11 (28.2)  14 (35.9)  0 | 83 (28.5)  98 (33.7)  110 (37.8)  2 | 0.521 |
| Autoimmune Disease (%)  No  Yes | 104 (92.0)  9 (8.0) | 20 (95.2)  1(4.8) | 124 (92.5)  10 (7.5) | 0.952 | 222 (87.4)  32 (12.6) | 34 (87.2)  5 (12.8) | 256 (87.4)  37 (12.6) | 1.000 |
| DM (%)  No  Yes | 97 (85.8)  16 (14.2) | 18 (85.7)  3 (14.3) | 115 (85.8)  19 (14.2) | 1.000 | 229 (90.2)  25 (9.8) | 34 (87.2)  5 (12.8) | 263 (89.8)  30 (10.2) | 0.774 |
| Hyperlipidemia (%)  No  Yes | 92 (81.4)  21 (18.6) | 13 (61.9)  8 (38.1) | 105 (78.4)  29 (21.6) | 0.088 | 209 (82.3)  45 (17.7) | 30 (76.9)  9 (23.1) | 239 (81.6)  54 (18.4) | 0.561 |
| Statin user (%)  No  Yes | 103 (91.2)  10 (8.8) | 15 (71.4)  6 (28.6) | 118 (88.1)  16 (11.9) | **0.028** | 240 (94.5)  14 (5.5) | 35 (89.7)  4 (10.3) | 275 (93.9)  18 (6.1) | 0.429 |
| Smoking (%)  None  Current or Ex-smoker  Missing data | 22(24.2)  69 (75.8)  22 | 4 (21.1)  15 (78.9)  2 | 26 (23,6)  84 (76.4)  24 | 1.000 | 180 (93.3)  13 (6.7)  61 | 30 (85.7)  5 (14.3)  4 | 210 (92.1)  18 (7.9)  65 | 0.237 |
| Drinking (%)  None  Mild to Moderate  Heavy  Missing data | 20 (29.0)  19 (27.5)  30 (43.5)  44 | 7 (46.7)  6 (40.0)  2(13.3)  6 | 27 (32.1)  25 (29.8)  32 (38.1)  50 | 0.092 | 86 (72.3)  29 (24.4)  4 (3.3)  135 | 16 (66.7)  6 (25.0)  2 (8.3)  15 | 102 (71.3)  35 (24.5)  6 (4.2)  150 | 0.531 |
| BMI [median, IQR]  Missing data | 22.8 [21.6; 24.5]  44 | 23.8 [23.1; 25.5]  6 | 23.4 [21.9; 25.0]  50 | 0.174 | 22.2 [20.2; 24.5]  133 | 21.5 [20.1; 22.9]  14 | 21.9 [20.1; 24.3]  147 | 0.149 |
| Cholesterol (mg/dL)  [median, IQR]  Missing data | 174.0  [155.0; 203.0]  44 | 184.0  [165.0; 204.5]  6 | 175.0  [156.0; 205.0]  50 | 0.338 | 181.0  [154.0; 204.0]  133 | 173.0  [151.0; 195.0]  14 | 181.0  [152.0; 203.0]  147 | 0.258 |
| FBS (mg/dL) [median, IQR]  Missing data | 97.0 [85.0; 107.0]  44 | 104.0 [96.0; 117.5]  6 | 98.0 [87.5; 108.5]  50 | 0.123 | 95.0 [84.0; 104.0]  133 | 92.0 [88.0; 99.0]  14 | 95.0 [85.0; 103.0]  147 | 0.841 |
| **At the time of GO diagnosis** |  |  |  |  |  |  |  |  |
| Autoimmune disease (%)  No  Yes | 101 (89.4)  12 (10.6) | 20 (95.2)  1 (4.8) | 121 (90.3)  13 (9.7) | 0.666 | 217 (85.4)  37 (14.6) | 34 (87.2)  5 (12.8) | 251 (85.7)  42 (14.3) | 0.965 |
| DM (%)  No  Yes | 86 (76.1)  27 (23.9) | 18 (85.7)  3 (14.3) | 104 (77.6)  30 (22.4) | 0.493 | 217 (85.4)  37 (14.6) | 29 (74.4)  10 (25.6) | 246 (84.0)  47 (16.0) | 0.128 |
| Hyperlipidemia (%)  No  Yes | 76 (67.3)  37 (32.7) | 11 (52.4)  10 (47.6) | 87 (64.9)  47 (35.1) | 0.288 | 192 (75.6)  62 (24.4) | 26 (66.7)  13 (33.3) | 218 (74.4)  75 (25.6) | 0.321 |
| Statin user (%)*  Non-statin user  Statin user | 98 (86.7)  15 (13.3) | 18 (85.7)  3 (14.3) | 116 (86.6)  18 (13.4) | 1.000 | 240 (94.5)  14 (5.5) | 36 (92.3)  3 (7.7) | 276 (94.2)  17 (5.8) | 0.861 |
| Thyroidectomy (%)  No  Yes | 112 (99.1)  1 (0.9) | 21 (100.0)  0 (0.0) | 133 (99.3)  1 (0.7) | 1.000 | 252 (99.2)  2 (0.8) | 38 (97.4)  1 (2.6) | 290 (99.0)  3 (1.0) | 0.863 |
| RAI (%)  No  Yes | 105 (92.9)  8 (7.1) | 21 (100.0)  0 (0.0) | 126 (94.0)  8 (6.0) | 0.450 | 239 (94.1)  15 (5.9) | 37 (94.9)  2 (5.1) | 276 (94.2)  17 (5.8) | 1.000 |
| Statin dose (mean, SD) | 38.0 ± 141.0 | 40.8 ± 95.2 | 38.4 ± 134.5 | 0.911 | 8.0 ± 40.8 | 23.4 ± 75.2 | 10.1 ± 47.0 | 0.220 |

GO, Graves’ orbitopathy; n, number; IQR, interquartile range; DM, diabetes mellites; BMI, body mass index; FBS, fasting blood sugar, DM, diabetes mellitus, RAI, radioactive iodine; SD, standard deviation.

*Statin user assessed during the follow-up periods. Bold type denotes statistical significance (*p* < 0.05).

Statin dose: mg, atorvastatin equivalent
